# Gold–Protein Composite Nanoparticles for Enhanced X-ray Interactions: A Potential Formulation for Triggered Release

**DOI:** 10.3390/pharmaceutics13091407

**Published:** 2021-09-04

**Authors:** Courtney van Ballegooie, Alice Man, Alessia Pallaoro, Marcel Bally, Byron D. Gates, Donald T. Yapp

**Affiliations:** 1 Experimental Therapeutics, BC Cancer, Vancouver, BC V5Z 4E6, Canada; cballegooie@bccrc.ca (C.v.B.); apallaoro@sfu.ca (A.P.); mbally@bccrc.ca (M.B.); 2 Faculty of Medicine, University of British Columbia, Vancouver, BC V6T 1Z3, Canada; 3 Department of Chemistry, Simon Fraser University, Burnaby, BC V5A 0A7, Canada; bgates@sfu.ca; 4 Department of Anesthesiology, Pharmacology and Therapeutics, University of British Columbia, Vancouver, BC V6T 1Z3, Canada; alice.man@alumni.ubc.ca; 5 Faculty of Pharmaceutical Sciences, University of British Columbia, Vancouver, BC V6T 1Z3, Canada

**Keywords:** nanomedicine, chemo-radiotherapy, Zein nanoparticles, microfluidics

## Abstract

Drug-delivery vehicles have been used extensively to modulate the biodistribution of drugs for the purpose of maximizing their therapeutic effects while minimizing systemic toxicity. The release characteristics of the vehicle must be balanced with its encapsulation properties to achieve optimal delivery of the drug. An alternative approach is to design a delivery vehicle that preferentially releases its contents under specific endogenous (e.g., tissue pH) or exogenous (e.g., applied temperature) stimuli. In the present manuscript, we report on a novel delivery system with potential for triggered release using external beam radiation. Our group evaluated Zein protein as the basis for the delivery vehicle and used radiation as the exogenous stimulus. Proteins are known to react with free radicals, produced during irradiation in aqueous suspensions, leading to aggregation, fragmentation, amino acid modification, and proteolytic susceptibility. Additionally, we incorporated gold particles into the Zein protein matrix to create hybrid Zein–gold nanoparticles (ZAuNPs). Zein-only nanoparticles (ZNPs) and ZAuNPs were subsequently exposed to kVp radiation (single dose ranging from 2 to 80 Gy; fractionated doses of 2 Gy delivered 10 times) and characterized before and after irradiation. Our data indicated that the presence of gold particles within Zein particles was correlated with significantly higher levels of alterations to the protein, and was associated with higher rates of release of the encapsulated drug compound, Irinotecan. The aggregate results demonstrated a proof-of-principle that radiation can be used with gold nanoparticles to modulate the release rates of protein-based drug-delivery vehicles, such as ZNPs.

## 1. Introduction

Chemotherapy and radiation therapy are potent treatment modalities for cancer, but delivering curative doses to the tumor is often precluded by chemo- or radiotoxicity to normal tissue. Delivery systems that concentrate drug or X-ray effects to the tumor (e.g., liposomes or volumetric modulated arc therapy (VMAT), respectively) are thus of significant therapeutic interest. For example, head and neck (H&N) cancer patients with unresectable disease are treated with X-rays and cisplatin (CPT) concurrently to take advantage of CPT/X-ray synergy for improved tumor control [1,2]. Concurrent chemo-radiation treatments show benefit in tumor control in H&N patients, wherein adding high-doses of CPT to radiation improved overall survival. Unfortunately, the systemic toxicity of CPT (e.g., kidney and nerve damage) is often dose-limiting [2,3]. Conceptually, there is interest in expanding concurrent chemo-radiation to other tumor sites (e.g., pancreatic cancer, glioma), but effective strategies to mitigate chemo- and radiotoxicity to normal tissue have yet to be developed.

One approach to limit toxicities would be to use radiation to trigger the release of a drug from nanoparticle drug carriers that accumulate in the tumor [4]. Drug release from the particles would then be preferentially modulated within the tumor, leading to high intratumoral drug concentrations and limiting potential negative cytotoxic effects of the drug outside the disease site. Selecting drugs that have synergistic effects with radiation could also potentiate the effects of radiation and potentially provide tumor control with lower doses of radiation, chemotherapy, or both [5,6].

Innovations in gantry design of radiation therapy (RT) machines to maximize delivery of high radiation doses safely to the tumor are close to their physical limits, but the variety of drug-delivery systems, some of which are already clinically used, could be modified to release their contents only when exposed to radiation. Typically, the constituents of the delivery vehicle are designed to balance the encapsulation and release of its contents. Efficient encapsulation could mean the drug is trapped and is never released. Conversely, inefficient encapsulation results in materials that are “too leaky”, with the drug being released before the vehicle reaches its intended target. Zein, a hydrophobic protein, was identified as having high potential as an efficient delivery system whose release characteristics could be modulated by external beam radiation.

In this work, a protein-based matrix made from Zein was used to encapsulate gold nanoparticles (AuNPs) and a drug. This Zein delivery system effectively encapsulated the drug with minimal release in the absence of the triggering mechanism. However, when the hybrid particles were irradiated, the protein matrix was modified and degraded by the radiation–gold particle interactions to release the drug on-demand. Overall, this strategy would enable more precise drug delivery to the tumor, as only particles in the target local environment receiving radiation would release drugs. Spatially specific and triggered delivery of the drugs by radiation has the potential to reduce systemic toxicities and, ultimately, increase drug concentration levels within the tumor itself.

## 2. Materials and Methods

### 2.1. Materials

Zein from maize (Cat. 9010-66-6) was purchased from Sigma-Aldrich (St. Louis, MO, USA), as well as a 40% acrylamide/bis-acrylamide solution (Cat. A7802; St. Louis, MO, USA) and 2-mercaptoethanol (Cat. M3148; Japan). Gold (III) chloride hydrate (Cat. 520918; USA) MW = 393.83 g/mol and sodium citrate tribasic dihydrate (Cat. 71402; Belgium), molecular weight (MW) of 294.1 g/mol, were purchased from Sigma. The following items were purchased from Bio-Rad Laboratories (USA): tetramethylethylenediamine (TEMED) (Cat. 161-0800), ammonium persulfate (Cat. 161-0700), sodium dodecyl sulphate (SDS) (Cat. 161-0302), resolving buffer (Cat. 161-0798), stacking buffer (Cat. 161-0799), 10× tris/glycine buffer (Cat. 161-0734), and 4× Laemmli sample buffer (Cat. 161-0747). A Pierce Silver Stain Kit (Cat. 24612) was purchased from ThermoFisher (Rockford, IL, USA). Ethyl alcohol anhydrous was purchased from Commercial Alcohols (Cat. P016EAAN; Brampton, ON, Canada). Acrodisc syringe filters (13 mm, 0.8 µm membrane) were purchased from Pall (Port Washington, NY, USA). Slip tip syringes for 3 mL (Cat. 309586) and 1 mL (Cat. 309659) volumes were purchased from Becton Dickinson (Franklin Lakes, NJ, USA). The 384-well flat-bottom black polystyrene TC-treated microplates were purchased from Corning (Cat. 3764; Germany). A protein ladder (Cat. PM007-0500S) was purchased from FroggaBio Scientific Solutions (Toronto, ON, Canada). Irinotecan was purchased Accord Health (DIN 02357585; Kirkland, QC, Canada). Zein NPs were synthesized using a Benchtop NanoAssemblr (Precision Nanosystems; Vancouver, BC, Canada). Samples were analyzed using a NanoBrook ZetaPALS (Holtsville, NY, USA) for dynamic light scattering (DLS). Ultraviolet visible spectroscopy (UV–vis) was performed using a Nanodrop ND-1000 spectrophotometer from Thermoscientific (Wilmington, DE, USA) and a Mandel CLARIOstar microplate reader from BMG LABTECH (Canada). Samples were shaken using a Thermomixer R from Eppendorf (Germany) unless otherwise specified. Centrifugation was performed on an Thermoscientific (Germany) Legend Micro 21R Centrifuge using Eppendorf tubes (VWR, Cat 20170-577; Radnor, PA, USA) unless otherwise specified. Samples were heated using an Accublock Digital Dry Bath from Labnet International (Edison, NJ, USA). Sonication was performed on a 2510 Ultrasonic Cleaner (40 kHz frequency) from Branson (Danbury, CT, USA). Gels were run on a Bio-Rad Mini-PROTEAN^®^ Tetra Cell (USA) and imaged using a Bio-Rad ChemiDoc MP Imaging system (USA). Electron microscopy (EM) techniques were performed using a FEI Tecnai Osiris S/TEM (Hillsboro, OR, USA) for all transmission electron microscopy (TEM) and scanning transmission electron microscopy (STEM) samples, while scanning electron microscopy (SEM) and energy-dispersive X-ray spectroscopy (EDX) images were acquired using an FEI Nova NanoSEM (Hillsboro, OR, USA), with all samples being prepared on a holey 300-mesh formvar/carbon-coated copper grid (Ted Pella, Cat. FCF-300; Hatfield, PA, USA). Cryogenic EM (Cryo-EM) samples were prepared using a Vitrobot from ThermoFisher (Hillsboro, OR, USA).

### 2.2. Gold Nanoparticle Synthesis

The 13 nm AuNP synthesis was adapted from Savchenko et al. [7]. Briefly, a 1 mM HAuCl_4_ solution was created by first combining 10 mL of deionized (DI) water (18.0 MΩ ultrapure water) with 197 mg of HAuCl_4_. This 50 mM solution was added to an Erlenmeyer flask containing 490 mL of DI water that was brought to a boil to achieve a 1 mM HAuCl_4_ solution. Then, 684 mg of sodium citrate dihydrate was dissolved in 25 mL of DI water and was rapidly added to the Erlenmeyer flask (approximately 4.5 mM). This solution was kept at a boil and stirred at 400 revolutions per minute (rpm) for an additional 30 min before the heat was turned off and the flask was left to cool. Once cooled, the AuNPs underwent further characterization (UV–vis, DLS, and EM) before use Figure S1 (Supplementary Materials). The AuNP stock was then stored at 4 °C and protected from light when in use.

### 2.3. Zein Purification

Zein was purified before use as previously reported by our lab [8]. Briefly, Zein (0.10 g) was suspended in 15 mL of anhydrous EtOH. The protein suspension was then stirred overnight (400 rpm at 4 °C). Afterwards, stirring was stopped and the insoluble Zein was allowed to settle. The supernatant was removed, and the collected Zein was resuspended in anhydrous EtOH again. This process was repeated twice. After the washes, the final solution of Zein was in 60% (*v*/*v*) EtOH. This purified Zein was filtered using a 0.8 µm syringe filter and was then ready for use in further experiments. All Zein stocks were kept at 4 °C and used within 2 weeks of production of their final suspension.

### 2.4. Zein Nanoparticle and Zein–Gold Hybrid Nanoparticle Synthesis and Characterization

Zein nanoparticle (ZNP) and Zein–gold hybrid nanoparticle (ZAuNP) synthesis procedures were adapted from a protocol previously reported by our lab [8]. Briefly, the purified Zein (0.10 g) that was dissolved in 10 mL of 60% (*v*/*v*) EtOH and filtered, as detailed in Section 2.3, was subsequently loaded into a syringe (1.0 mL) as the organic phase in the first inlet channel in the microfluidics device. Its counterpart, the aqueous phase (in the presence or absence of citrate-coated 1 nM 13 nm AuNPs suspended in water) was loaded into a 3.0 mL syringe in the second inlet channel. The excess citrate was removed from the AuNPs by spinning down the sample (20,000× *g* for 20 min at 4 °C) and resuspending it in water by sonicating cycles of 5 s on, 5 s off until the AuNP pellet was no longer seen. The AuNP sample was then diluted to the appropriate concentration using UV–vis (molar extinction coefficient of 3.67 × 10^8^ M^−1^cm^−1^) at an absorbance of 520 nm. The sample was run at a total flow rate of 2 mL/min and a relative flow rate of 3:1 (aqueous to organic). The samples were then collected at the microfluidics chip’s outlet channel. Each run was programmed to discard the first 0.3 mL of the sample and the last 0.1 mL of the sample to ensure that the variability in fluid dynamics at the start and end of the synthesis run did not affect the sample.

### 2.5. Zein Nanoparticle and Zein–Gold Hybrid Nanoparticle Irradiation

First, 200 µL of the ZNP and ZAuNP was put aside as our nonirradiated control. An additional 200 µL of each sample was added to a 96-well plate and irradiated. Irradiation was performed at the BC Cancer Research Institute using an X-ray tube with a tungsten anode operated at 300 kVp and 10 mA. The radiation was filtered through a 2 mm Al plate, and the dose rate in the sample plane was approximately 0.06 Gy/s. The irradiation time was set to deliver doses of 2, 10, 20, 40, or 80 Gy. Additionally, some samples underwent a fractioned dosing scheme whereby 2 Gy was administered every 5 min until a total radiation exposure of 20 Gy was delivered. Samples then underwent further analysis (DLS, UV–vis, sodium dodecyl sulphate–polyacrylamide gel electrophoresis (SDS-PAGE), and EM).

### 2.6. SDS-PAGE

A 15% (*v*/*v*) acrylamide running gel with 5% (*v*/*v*) stacking gel was prepared for the SDS-PAGE. The ZNP and ZAuNP samples (10 µL) were combined with a 3:1 Laemmli sample buffer containing beta-mercaptoethanol (BME) and warmed at 100 °C for 10 min. Then, 5 µL of the protein ladder and 10 µL of the samples were loaded into each of the wells. A glycine-tris buffer with 0.1% (*w*/*v*) SDS was used as the running buffer, and the samples were run at 200 V for 40 min. Gels were then stained using a Pierce Silver Stain Kit. Briefly, the gels underwent two 5 min washes in DI water. The gels were then fixed in a solution containing 30% (*v*/*v*) EtOH and 10% (*v*/*v*) acetic acid solution 2 times for 15 min each. Subsequently, the gels were washed 2 times in a 10% (*v*/*v*) EtOH solution for 5 min and then washed 2 times with DI water for 5 min each. Gels were sensitized for 1 min and then washed twice for 1 min using DI water. Following the sensitization and wash period, the gels were stained with the staining solution for 30 min. After staining, the gels underwent two 20 s washes in DI water and were allowed to develop for 2–3 min. Once the bands appeared, the reaction was stopped with a 5% acetic acid wash. The gels were washed in this solution for 10 min and then stored in DI water until they were imaged under a transilluminator (0.3 ms exposure) for further analysis.

### 2.7. UV–Vis Aromaticity and Turbidity

Aromaticity (OD 280 nm) and turbidity (OD 340 nm) were determined at room temperature (23 ± 1) °C using UV–vis. When proteins are exposed to wavelengths between 270–290 nm, their aromatic compounds (such as the tryptophan and tyrosine residues) absorb strongly within these wavelengths. Aromaticity was measured at 280 nm using the Nanodrop at an undiluted concentration of the ZNP and ZAuNP samples. Turbidity was measured using a modified protocol by Larson et al. in which 20 μL of sample was combined with 20 μL of water and then added to a clear-bottom 384-well plate [9]. The sample was shaken at 300 rpm for 30 s, and the absorbance was read at 340 nm in a CLARIOstar plate reader.

### 2.8. Irinotecan Loading and Triggered Release

A final concentration of 50 µg/mL of Irinotecan was incorporated in the Zein “organic phase” of the microfluidics coprecipitation synthesis. The Zein–Irinotecan suspensions were sonicated for 5 cycles of 5 s on and 5 s off before they were shaken for 1 h [400 rpm at (23 ± 1) °C] before use in the microfluidics system. Irinotecan-loaded ZNPs with or without AuNPs were synthesized using the conditions listed in Section 2.4. Then, 400 µL of the samples were washed two times using an Amnicon Ultra 0.5 mL 50 k ultracentrifugation filter at 1000× *g* for 70 min at 4 °C. The supernatants that did not contain Zein were analyzed using UV–vis absorbance at 370 nm in order to determine the amount of free Irinotecan to calculate the encapsulation efficiency (EE). The EE was then determined using the following formula:

EE = ((Total Irinotecan − Free Irinotecan)/Total Irinotecan) × 100%(1)

Samples were then brought to a final volume of 200 µL using DI water, then 100 µL of the Irinotecan-loaded ZNPs and ZAuNPs samples were irradiated at 20 Gy as described above. All samples were subsequently spun down (1000× *g* for 30 min) using a centrifuge tube, and 100 µL of the supernatant, which did not contain the Zein, was collected from each sample and evaporated at 50 °C overnight. After the evaporation, samples were resuspended in 50 µL of 100% EtOH. Then, 40 µL of each sample was analyzed on the plate reader for Irinotecan and compared against an Irinotecan standard curve to determine release. Conditions for the plate reader were: 300 rpm with double orbital shaking before reads, a gain of 2200, a focal height of 3.4 mm, an excitation wavelength of 365 nm with a 20 nm slit width, and an emission wavelength of 450 nm with a 10 nm slit width. During the scan, 40 flashes per well were read from the bottom optic, with 0.1 s settling time in between reads.

### 2.9. SEM, STEM, EDX, and Cryo-Electron Microscopy

ZNP and ZAuNP samples were diluted to a 10 times lower concentration by adding DI water immediately prior to microscopy sample preparation. The sample (2 µL) was spotted on the copper grid and dried under vacuum (1–2 h). The samples were imaged immediately after using a Nova NanoSEM or a STEM Osiris (200 kVp acceleration voltage, bright field, high-angle dark field, and energy-dispersive X-ray). For cryo-electron microscopy (Cryo-EM), samples were used at their initial concentration. Preparation of the plunge-frozen samples for Cryo-EM followed the protocol developed by Grassucci et al. [10]. Briefly, 2 µL of sample was added to a plasma cleaned holey carbon copper grid. The sample was kept in a 100% relative humidity, 6 °C climate before being blotted for 1 s using a Vitrobot. Directly following the blotting, the sample was plunge-frozen in liquid ethane. The sample was kept at temperatures below devitrification by using precooled tools and holders. Once loaded into the cryo-grid sample holder, the sample was immediately imaged using TEM.

### 2.10. Statistics and Image Processing

Data was reported as the mean, with the error bars representing the standard deviation (SD). Data sets had their normality tested using a Shapiro–Wilk test and/or a Quantile–Quantile plot when small sample sizes were present (*n* = 3). Equal variance was tested using a Brown–Forsythe test. Normally distributed data with equal variance then underwent an analysis of variance (ANOVA) test. If the data was not normally distributed, then a nonparametric Kruskal–Wallis test, was employed. Multiple comparison tests, such as Tukey’s tests, were utilized to identify differences between sample means. Statistics were analyzed using GraphPad Prism 9. Statistical significance was declared at the following probability levels: * *p* < 0.05, ** *p* < 0.01, *** *p* < 0.001, **** *p* < 0.0001, and ns for non-significant. Image processing to determine ZNP and ZAuNP aggregation was performed on the SDS-PAGE images using ImageJ. Briefly, the integrated density (ID) of sample above the 25 kDa protein marker was measured. The ID of the sample was then normalized to the ID of the lane. Finally, the ID of the experimental sample lane was then divided by the ID of the control lane to determine the fold change.

## 3. Results

### 3.1. Synthesis and Characterization of Zein and Zein–Gold Hybrid Nanoparticles

**Figure 1:**
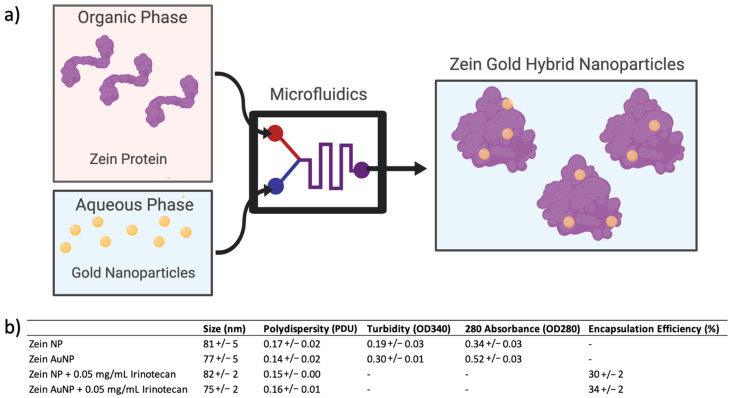
(**a**) Schematic representation of Zein–gold hybrid nanoparticles (ZAuNPs); (**b**) standard characteristics of Zein-only nanoparticles (ZNPs) and ZAuNPs including size, polydispersity index, turbidity, and 280 nm absorbance and loading characteristics of Irinotecan formulations.

**Figure 2:**
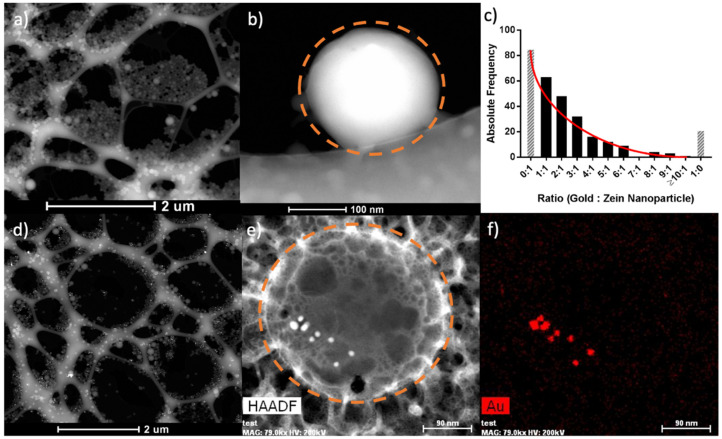
Representative electron microscopy images of Zein nanoparticles (ZNPs) and Zein–gold hybrid nanoparticles (ZAuNPs) and the number of associated gold nanoparticles identified per ZAuNP. (**a**) A low-magnification dark field scanning transmission electron microscopy image of Zein NPs. (**b**) A high-magnification dark field scanning transmission electron microscopy image of a Zein NP. The dashed orange line indicates the outline of the ZNP. (**c**) A graph depicting the number of gold nanoparticles associated per ZAuNP. The black bars represent hybrids, while the gray hashed bars represent either Zein- or gold-only nanoparticles. (**d**) A low-magnification dark field scanning electron microscopy image of ZAuNPs. (**e**) A high-magnification dark field scanning electron microscopy image of ZAuNPs. The dashed orange line indicates the outline of the ZAuNP. (**f**) An energy-dispersive X-ray spectroscopy image of (**e**), confirming the presence of gold nanoparticles.

Following the procedure to synthesize the ZNPs and ZAuNPs (depicted in Figure 1a), the final product was analyzed using DLS, UV–vis, and EM (Figure 1b and Figure 2). ZNPs were optimized formerly for the microfluidics system as described previously [8]. Briefly, the total flow rate, relative flow rate, solvent concentration and type, and protein concentration were optimized to achieve sub-100 nm nanoparticles reproducibly. The addition of the 1 nM 13 nm AuNPs to the aqueous phase to form the ZAuNP hybrids had little effect on the size and polydispersity (e.g., the level of heterogeneity of the sizes of the nanoparticles), as seen in Figure 1b. Interestingly, the ZAuNPs displayed higher baseline turbidity and absorbance at 280 nm when their UV–vis characteristics were probed. This has been reported in the literature previously, and our results suggested alignment with this finding in that the incorporation of metallic nanoparticles, such as AuNPs, may influence the secondary and tertiary structure of the protein, thereby altering the absorption characteristics of the protein carrier [11,12].

**Figure 3:**
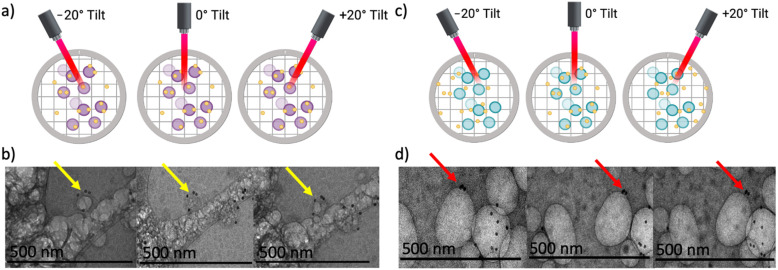
Tilting of the sample in cryogenic transmission electron microscopy (Cryo-TEM) to determine the association of particles. (**a**) A schematic of how hybrid particles would appear at each tilting angle used for the Cryo-TEM. (**b**) Cryo-TEM depicting gold nanoparticles (black) continuing to associate with Zein nanoparticles (dark grey) as the sample was tilted at −20°, 0°, and +20°, from left to right. (**c**) A schematic of how nonassociated particles would appear when the particles will not remain associated when changing the tilting angle. (**d**) Cryo-TEM depicting gold nanoparticles (black) disassociating with a bubble (light grey) as the sample is tilted at −20°, 0°, and +20°, from left to right.

The particles’ sizes, morphologies, and hybrid assemblies were further investigated using SEM, STEM, and Cryo-TEM. STEM and SEM micrographs of the ZNP and ZAuNP presented spherical nanoparticles as depicted in Figure 2a,b, respectively. The EDX micrograph of the ZAuNP hybrid nanoparticle confirmed that the incorporated metallic nanoparticles were composed of gold. To gain better understanding of the number of particle associations between the AuNPs and ZNPs when formulating the ZAuNP hybrids, 15 Cryo-TEM images were hand-counted to determine the ratio of AuNPs to ZNPs in each ZAuNP hybrid. In total, 275 ZNPs and 532 AuNPs were identified within these images. It was found that the number of AuNPs incorporated with ZNPs had an exponential decrease, with a 1:1 ratio being the most common hybrid population. It should be noted that approximately 30% of ZNPs were found to be unassociated with any AuNPs. Additionally, our data highlighted that the majority of AuNPs were incorporated into ZNPs, with less than 5% of AuNPs identified as being unassociated with Zein. The association of the AuNPs with the ZNPs was further confirmed by tilting the stage of the TEM, as demonstrated in Figure 3a,c. Figure 3b,d depict a Cryo-TEM image of associated and unassociated AuNPs, respectively. The ZAuNP hybrid appears to be associated with the AuNP at every tilting angle. It should be noted that the Zein experienced damage when imaged at a high magnification for multiple micrographs as evidence of the beam damage formed in the particle itself. Alternatively, Figure 3d displays an AuNP that appears to be associated with a bubble at a −20° tilt angle, but subsequently appears to be unassociated at both 0°and +20° tilting angles.

#### 3.1.1. Influence of X-rays on Zein and Zein–Gold Hybrid Particle Characteristics

**Figure 4:**
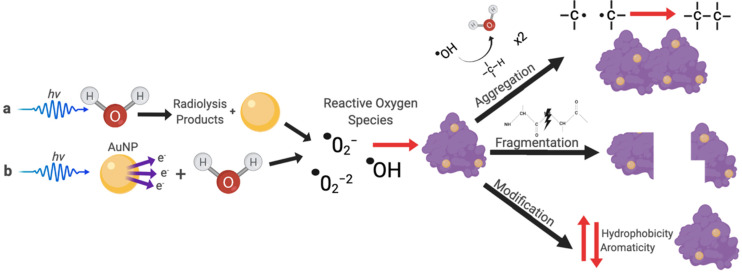
A schematic depicting the possible interactions of Zein with X-rays (*hv*) either: (**a**) directly through water radiolysis or (**b**) indirectly through interactions with gold nanoparticles (AuNPs).

One of the major effects of radiation is reactive oxygen species (ROS) generation through the radiolysis of water. The addition of gold, such as in AuNPs, which is electron dense, can enhance interactions with ionizing radiation. This ultimately leads to a greater production of ROS [4]. While ROS enhancement has been extensively studied with AuNPs, the lifespan of some of the ROS species is extremely short, making it difficult to track the process in real time when using complex samples [13]. However, physical and chemical characterization of the protein could alternatively reveal the possible downstream effects of such interactions. Figure 4 highlights some of the known assessable consequences of ROS interactions with proteins, including aggregation, fragmentation, and chemical modification (such as changes in aromaticity and hydrophobicity), which have been extensively used to characterize protein–radiation interactions [14,15,16,17,18]. These downstream effects were further probed using UV–vis, DLS, SDS-PAGE, and EM in our system.

**Figure 5:**
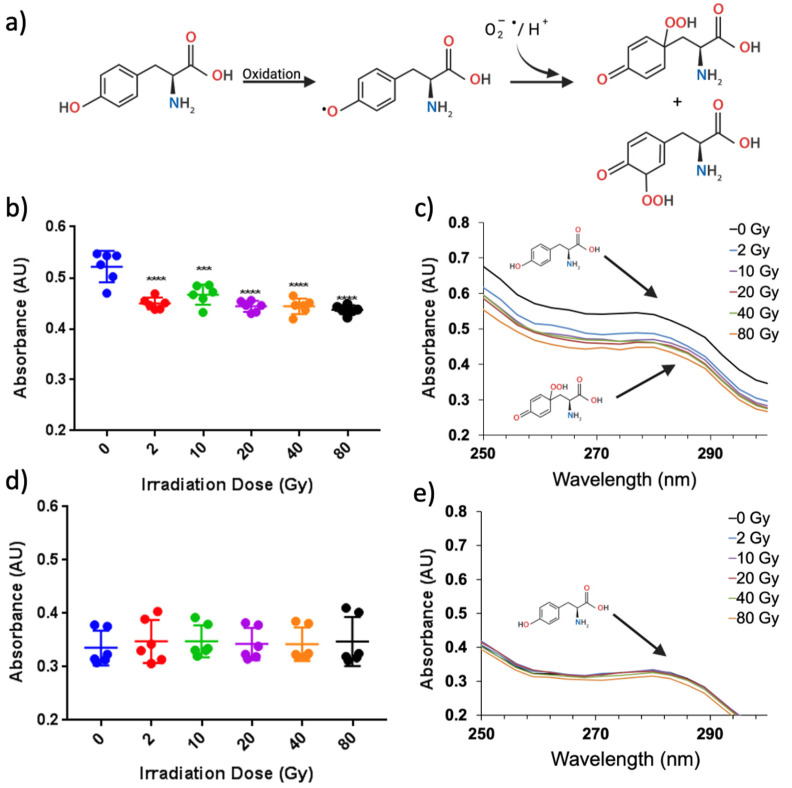
Suggested damage to the aromatic groups for irradiated Zein hybrid particles only: (**a**) a schematic illustration of a chemical reaction on the amino acid tyrosine adapted from Davies [23] which can undergo further reactions; (**b**) OD 280 nm of Zein–gold hybrid nanoparticles at varying irradiation doses; (**c**) representative absorbance spectra of Zein–gold hybrid nanoparticles at each dose in (**b**); (**d**) OD 280 nm of Zein nanoparticles at varying irradiation doses; (**e**) representative absorbance spectra of Zein nanoparticles at each dose in (**d**). *** *p* < 0.001, **** *p* < 0.0001.

**Figure 6:**
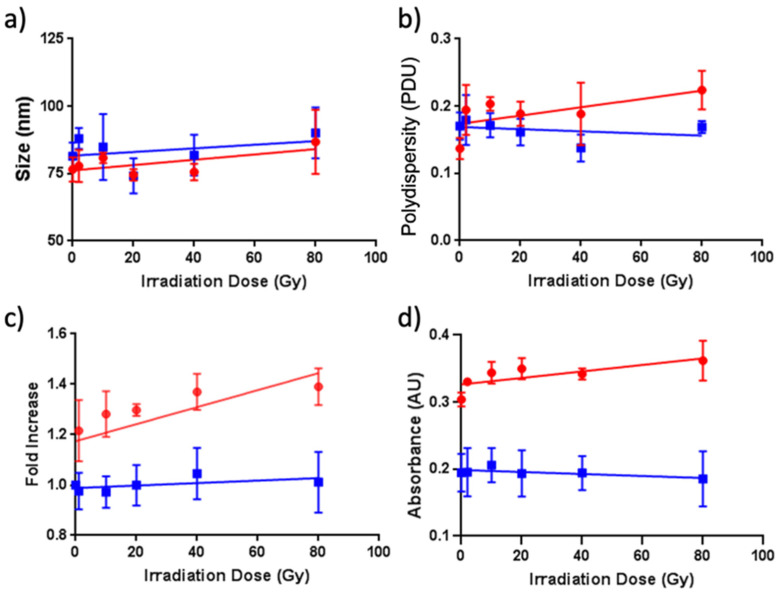
Characteristics of Zein-only nanoparticles (ZNPs; blue squares) and Zein–gold hybrid nanoparticles (ZAuNPs; red circles) at varying X-ray doses: (**a**) the size of ZNPs and ZAuNPs (dynamic light scattering results); (**b**) the polydispersity of ZNPs and ZAuNPs; (**c**) the level of aggregation of ZNPs and ZAuNPs (SDS-PAGE results); (**d**) the turbidity of ZNPs and ZAuNPs.

UV–vis was used to evaluate both the aromaticity and turbidity of the ZNPs and ZAuNPs after radiation exposure. X-ray irradiation caused a decrease in the absorbance found at 280 nm (Figure 5b,c) when AuNPs were present. While there was a significant decrease when comparing the nonirradiated control with each of the irradiated samples, there was no significant trend of decreased absorptivity at 280 nm with increasing irradiation dose (Figure S2). This finding was not seen in the ZNP samples irrespective of the irradiation dose, as depicted in Figure 5d,e. Interestingly, there have been many studies describing a protein’s aromatic absorbance when exposed to different sources of irradiation (UV, ɣ-rays, and X-rays). Within these studies, the absorbance at 280 nm relied heavily on the properties of the protein itself, as well as the surrounding environment, with some proteins displaying lower 280 nm absorbances at low Gy irradiation doses [14,17,18]. Interestingly, the presence of metallic nanoparticles has indeed been shown to influence the net decrease in 280 nm absorbance when irradiated, which is in alignment with our results [11,19]. Next, the turbidity of the sample was assessed at a 320 nm wavelength. This wavelength is commonly used for proteins, and it has been found to be an appropriate wavelength to be used for microaggregate assessment, as proteins typically display low absorptive properties at a 320 nm wavelength [20]. In Figure 6d, it can be seen that absorbance at 320 nm increased for ZAuNPs with an increasing irradiation dose. This was found to be statistically significant at every irradiation dose level relative to the nonirradiated control samples, thereby suggesting that microaggregation of the protein nanoparticles may be taking place (Figure S3). Increases in turbidity have commonly been attributed to the radiolysis of proteins, which results in changes in protein conformation accompanied by the dehydration, scission, and/or aggregation of the protein [14]. This increase in turbidity was not observed with ZNP samples irrespective of the irradiation dose (Figure 6d); moreover, there was no statistical difference identified between any of the irradiated test conditions of the ZNP and ZAuNP samples (Figure 6b). Our results suggested that the presence of AuNPs may play a role in promoting Zein turbidity when exposed to X-rays.

While turbidity is one method to evaluate the potential presence of aggregation for ZNPs and ZAuNPs, DLS and SDS-PAGE can provide additional clarity regarding the potential impact of AuNP presence during X-ray exposure. The irradiation of proteins can involve both inter- and intra-crosslinking, resulting from a variety of reactions, including carbon–carbon crosslinking of the backbone, aromatic crosslinking, and even the formation of new disulfide bridges between cystines [21,22,23,24]. For Zein in particular, aromatic crosslinking (such as by tyrosine formation) is anticipated to be the predominant mechanism if crosslinking is present due to the oxygen-rich environment in which these studies were conducted, as well as the negligible amounts of sulfur-containing amino acids found in Zein [21,25,26]. In order to further evaluate the presence of crosslinking, SDS-PAGE was performed. Figure 6c shows that there was an increase in aggregation upon X-ray irradiation when AuNPs were present. This finding was evident and significant at every irradiation dose that was evaluated, further suggesting that crosslinking could be taking place (Figure S3). Alternatively, there was found to be no association in aggregation when using SDS-PAGE to evaluate ZNPs exposed to equivalent levels of irradiation. To better understand this effect on a macro scale, the sample was further subjected to DLS. While there was an overall statistically significant increase in size with increasing irradiation dosage for the ZAuNPs, the absolute size change was very small (Figure 6a). This, however, was accompanied by a significant change in polydispersity, increasing from (0.14 ± 0.02) PDU to (0.22 ± 0.03) PDU. Taken together, these findings may suggest that competing mechanisms (aggregation and fragmentation) were taking place simultaneously, with the net effect resulting in only a very modest increase in particle size and a significant increase in the polydispersity, as reported previously in the literature [21]. It should also be noted that there were no obvious morphological changes between the irradiated and nonirradiated samples for the ZNP and ZAuNPs seen using STEM (Figure S4). Lastly, when evaluating all the characteristics described, we found no significant difference when the radiation was applied as a single dose or 2 Gy fractions (Figures S8 and S9), suggesting that these formulations may be applicable for use over longer timeframes.

#### 3.1.2. Triggered Release

**Figure 7:**
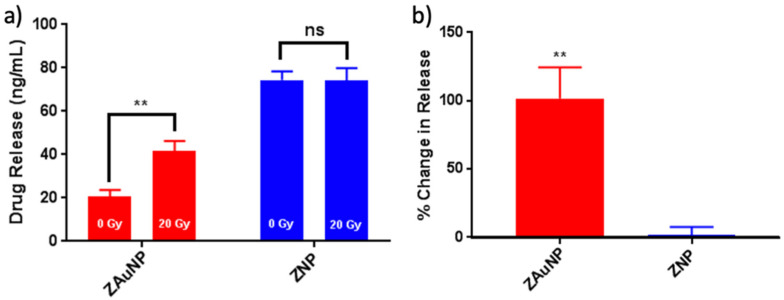
Irinotecan release of Zein nanoparticle (ZNP) and Zein–gold nanoparticle hybrid (ZAuNP) 50 µg/mL formulations. (**a**) Concentration of drug release of ZNP (blue) and ZAuNPs (red) before (left bar graph) and after (right bar graph) exposure to 20 Gy. (**b**) Percent change in Irinotecan release for ZNP and ZAuNP samples when exposed to 20 Gy. ** *p* < 0.01, ns for non-significant.

The EE of ZAuNPs and ZNPs were calculated as (34 ± 2) % and (30 ± 2) % for the 50 µg/mL formulations, respectively (Figure S6), as described in the Materials and Methods section [27]. While the loading of Irinotecan into ZNP and ZAuNP has not yet been reported, the more hydrophobic and potent analogue (SN38) has achieved higher EEs in ZNP samples [28]. Characteristics, including the size and polydispersity, of the nanoparticles post-Irinotecan loading showed very little change (Figure 1b). Additionally, no gross changes to the ZNPs’ or ZAuNPs’ morphology were identified (Figure S5). Samples then underwent irradiation at 20 Gy, as seen in Figure 7a, and only the ZAuNPs displayed triggered-release characteristics. The ZAuNP samples had an average difference in Irinotecan release of (26.4 ± 4.7) ng/mL, resulting in a 101% increase in release (Figure 7b). The ZNP samples had an average difference in Irinotecan release of (−0.20 ± 5.6) ng/mL, resulting in a 0% increase in release (Figure 7b). It should also be noted that freely suspended Irinotecan did not demonstrate a decrease in fluorescence upon irradiation (Figure S7).

## 4. Discussion

NP-based drug-delivery systems have been investigated over the past few decades to improve the efficacy, bioavailability, and circulation half-life of small-molecule drugs [29]. Recently, there is growing interest in using external stimuli, such as RT, to trigger drug release from NPs to enable usage of highly stabilized systems that will not release their cargo during circulation [4]. This strategy would be useful for high-dose chemo-radiotherapy treatments, such as those used in H&N cancer or other cancers undergoing further investigation using this treatment strategy by mitigating systemic toxicities and improving quality of life and/or treatment compliance [1,2,3].

Zein, in particular, is an attractive NP candidate since the protein: (1) is biocompatible, is formulated in nontoxic conditions, and has been given a generally recognized as safe (FDA GRAS) status; (2) is derived from the endosperm of corn, and is therefore a renewable and abundant resource; (3) contains a large number of reactive moieties available for functionalization; and (4) is amphiphilic and hydrophobic in nature, and possesses the ability to form NP structures [30,31]. While ZNPs have not yet been implemented in the clinic, Ethibloc^®^, a solubilized formulation of Zein, is clinically approved in Canada and has been investigated for the treatment of epistaxis, aneurysmal bone cysts, and lymphangiomas. In long-term studies, Ethibloc^®^ was found to be a safe and effective treatment for lymphangiomas and aneurysmal bone cysts, with minor short-term complications and no long-term side effects [32,33,34,35,36,37]. Although Ethibloc^®^ is a non-NP formulation of Zein, it is nonetheless a highly encouraging display of the protein’s biocompatibility, and complements the clinical potential of ZNPs alongside the large number of emerging chemotherapy-based ZNP formulations published in recent years.

While Zein has been established as a reliable carrier for chemotherapeutic compounds, the ability to use X-rays to trigger drug release from its matrix deserves further investigation. Our research suggests that the presence of AuNPs in Zein protein matrixes has a significant impact on the carrier’s characteristics when exposed to X-rays, and displays potential as a method to trigger drug release. Gold (Z = 79), the element composing our AuNPs, contains a large number of electrons, which can enhance the probability of interactions occurring between ionizing radiation and the atoms. The notion of using high-atomic-number materials to increase the dose given to a tumor during RT was first investigated over 25 years ago using iodine on cultured cells [38]. This concept was later expanded to an in vivo setting, when Santos Mello et al. directly injected iodine intratumorally in mice, in combination with RT, to suppress the growth of tumors [39]. A shift towards using AuNPs as radio-enhancers occurred due to their higher relative atomic number and biocompatibility [40,41]. Mechanistically, radiation energy can eject outer shell electrons from the gold directly, or be deflected by an electron, which itself is ejected. Resulting vacancies in lower electron shells can be filled by electrons from outer shells, which results in the ejection of Auger electrons during the process [42]. The ejected electrons from AuNPs deposit energy along their trajectory, radiolyze water, and initiate a cascade of reactions to produce ROS, such as the hydroxyl radical and hydrogen peroxide [43]. This phenomenon is thought to potentiate the cell-killing effects of radiation in vitro [42,44,45]. Additional studies have shown that ROS production and radiolysis of water is more efficient for distances close to the AuNPs compared to locations further away in the solution [41,46]. It was reported that water molecules around AuNPs interact with the gold surface via hydrogen bonding, rendering the H-OH bond more vulnerable to reaction with ROS present in the solution, which ultimately increases production of the hydroxyl radical [41]. Irradiating AuNPs embedded in a protein matrix, such as Zein particles, will thus likely generate high, localized concentrations of ROS. This increase of ROS production within the Zein matrix may explain the change in characteristics that were observed in the ZAuNP hybrids when irradiated.

Although Zein X-ray irradiation has not been well studied, ɣ-ray irradiation of Zein films has been extensively researched. In these studies, properties including aggregation, fragmentation, and modification of the Zein were characterized. These properties were investigated using SDS-PAGE, solubility, water vapor permeability (WVP), and water contact angle (WCA). Similar to our SDS-PAGE findings, Zhang et al. and Lee et al. identified a consistent decrease in band intensity for the alpha Zein constituents with increasing irradiation exposure [47,48]. Interestingly, in that same study by Lee et al., the authors also identified an increase in water solubility with increasing irradiation exposure of the films. This combination of findings led the authors to hypothesize that the Zein films were experiencing both crosslinking and fragmentation when exposed to radiation [47]. The hypothesis, which postulates that both fragmentation and crosslinking were occurring simultaneously, is in alignment with our DLS evaluations, which demonstrated that the ZAuNP particle size did not substantially increase, while the polydispersity of the system did increase. Next, studies by Soliman et al. evaluated the interaction of Zein films with water using WVP and WCA. In these studies, the WCA increased while the WVP decreased with increasing irradiation doses. Both WVP and WCA rely on the hydrophilic nature of the material. These results suggest that increasing irradiation causes an increase in hydrophobicity [49,50]. While these studies were performed on Zein films, similar findings for albumin nanoparticles also have been reported [13]. These findings may also explain our observed increase in turbidity with increasing irradiation. Should Zein particles become too hydrophobic, they may destabilize and ultimately “crash out” of solution, thereby increasing the turbidity of the sample.

Combining Zein with AuNPs to form ZAuNP hybrids is a relatively new research area, with only a few papers having been published to date [28,51,52,53,54]. While AuNPs have been combined with Zein in different forms (such as films, fibers, and nanoparticles), only one paper, to our knowledge, has considered the incorporation of AuNPs in ZNPs for tumor-killing purposes. In a study by Chauhan et al., near-infrared light (NIR) was used for tumor ablation, thereby capitalizing on AuNPs’ surface plasmon resonance at these wavelengths to induce localized heating. Clinically, however, NIR has limited treatment applicability in cancer, since NIR has restricted penetration depth in the body, and would almost certainly have difficulty reaching deeply situated tumors. X-rays, on the other hand, do not have penetration-depth limitations, and can be modulated to deposit high doses with precision at the tumor site. Additionally, while NIR was used for tumor ablation, the consideration of using the localized heating as a form of triggered release was not considered. Triggered drug release, however, has been studied in ZNP hybrid systems using iron oxide complexes and an external magnetic triggering device, thereby further demonstrating its potential as a triggered-release system [27]. In our drug-release experiments, it was observed that only the ZAuNP formulations exhibited triggered release. It was also identified that the ZNP–Irinotecan formulation had a higher baseline release than our ZAuNP–Irinotecan formulation. This observation could be due to either experimental artifacts and/or to the different characteristics of the systems themselves. Disparities in the characteristics of the ZNP and ZAuNP samples may explain the observed differences in their baseline release characteristics. Two of the Zein characteristics that could be influenced due to the presence of the AuNPs are its zeta potential, as well as its secondary and tertiary profiles. Zein, at physiological pH, has a negative charge at approximately −35 mV; however, due to the presence of negatively charged AuNPs, there has been an observed further decrease in zeta potential when combining the two nanoparticles [30,52]. It has often been observed that changing the charge of the components, such as the small molecule’s charge, can influence the release characteristics of the system [27,55]. Additionally, it has been identified that the incorporation of nanoparticles, including gold–silica nanoparticles in Zein, modifies the secondary and tertiary characteristics of the protein. Specifically, it has been noted that the alpha helical content of proteins decrease in the presence of metallic nanoparticles [11,12]. Due to the presence of AuNPs in the ZNPs, the new configuration that the Zein adopts could change the release characteristics, such as a decrease in swelling or porosity, as observed in Zein AuNP films [56]. If the AuNPs hindered the swelling ability of the ZAuNPs, this could have altered the release characteristics, as seen in many hydrogel systems, thereby leading to a slower release of the Irinotecan [57]. Overall, the incorporation of AuNPs makes a direct comparison of the nanoparticles’ EE and release rates difficult due to the change in the Zein’s structural conformation. Despite these speculations and potential limitations of the experiment, however, less than 0.5% of the calculated encapsulated payload was released for the both the ZNP and ZAuNP formulations when no radiation was present, suggesting that both had low baseline release of Irinotecan, and that only the ZAuNP formulation demonstrated triggered release.

## 5. Conclusions

Early detection of small tumors that have not spread, followed by surgery and adjuvant chemotherapy and radiotherapy, is the key to the successful treatment of many cancers. However, not all solid tumors can be removed surgically due to their proximity to critical normal structures or patient comorbidities. The only treatment options for these patients are RT and chemotherapy, or some combination of both. Chemo-radiotherapy, however, often results in non-negligible toxicities to the patient. This paper proposed utilizing the characteristics of Zein protein in order to design a composite AuNP hybrid particle for triggered drug release. By sequestering and releasing the drug at the site of the tumor, the toxicity profile of the chemo-radiotherapy could potentially lessen. The system would capitalize on the radio-enhancing capabilities of AuNPs that would, in turn, result in changes in the ZNP matrix, and subsequently enhance drug release. Radiation could thus be used to control drug release from ZAuNP hybrid particles selectively in a tumor as described above. Overall, we observed changes in our ZNP matrix, such as aggregation, fragmentation, and modification to the protein’s hydrophobicity and aromaticity, when AuNPs were present upon irradiation. These changes were measured using SDS-PAGE, UV–vis, DLS, and EM. Additionally, our study demonstrated proof-of-principle results of triggered drug release using a Zein–AuNP composite nanoparticle system in the presence of X-rays.

## Data Availability

Data can be made available upon request.

## Supplementary Materials

The following are available online at https://www.mdpi.com/article/10.3390/pharmaceutics13091407/s1, Figure S1: A histogram and TEM image of the 13 nm gold nanoparticles used to make ZAuNPs, Figure S2: A summary graph and spectra of the 280 nm absorbance at varying levels of irradiation, Figure S3: The individual measurements for each standard condition measured, Figure S4: STEM images of the nanoparticles at irradiation levels of 0 and 80 Gy, Figure S5: STEM images of the nanoparticles loaded with Irinotecan, Figure S6: Loading characteristics of the Zein and hybrid nanoparticles, Figure S7: Irinotecan fluorescent characteristics at different levels of irradiation, Figure S8: Standard characteristics of the hybrid particles at 20 Gys administered as a single dose or fractioned, and Figure S9: Standard characteristics of the Zein particles at 20 Gys administered as a single dose or fractioned.

## Abbreviations

Analysis of Variance	(ANOVA)
Cisplatin	(CPT)
Cryogenic Transmission Electron Microscopy	(Cryo-TEM)
Deionized	(DI)
Dynamic Light Scattering	(DLS)
Electron Microscopy	(EM)
Gold Nanoparticle	(AuNP)
Generally Recognized as Safe	(GRAS)
Head and Neck	(H&N)
Integrated Density	(ID)
Molecular Weight	(MW)
Near-Infrared Light	(NIR)
Optical Density	(OD)
Reactive Oxygen Species	(ROS)
Revolutions per Minute	(rpm)
Radiation Therapy	(RT)
Scanning Electron Microscopy	(SEM)
Scanning Transmission Electron Microscopy	(STEM)
Sodium Dodecyl Sulphate	(SDS)
Sodium Dodecyl Sulphate–Polyacrylamide Gel Electrophoresis	(SDS-PAGE)
Tetramethylethylenediamine	(TEMED)
Transmission Electron Microscopy	(TEM)
Ultraviolet Visible Spectroscopy	(UV–vis)
Volumetric Modulated Arc Therapy	(VMAT)
Water Contact Angle	(WCA)
Water Vapor Permeability	(WVP)
Zein Nanoparticle	(ZNP)
Zein–Gold Hybrid Nanoparticle	(ZAuNP)
