# Supplementary material for: Gold–Protein Composite Nanoparticles for Enhanced X-ray Interactions: A Potential Formulation for Triggered Release
Source: Pharmaceutics. 2021 Sep 4;13(9):1407. doi: 10.3390/pharmaceutics13091407 (PMC8471296; doi:10.3390/pharmaceutics13091407)
Supplement: Supplementary file 1 [file pharmaceutics-13-01407-s001.zip › pharmaceutics-1325228-supplementary.pdf]

pharmaceutics

MDPI

Supplementary Material: Gold Protein Composite Nanoparticles for Enhanced X-ray Interactions: A Potential for Triggered Release

Courtney van Ballegooie, Alice Man, Alessia Pallaoro, Marcel Bally, Byron Gates and Donald T. Yapp

Table of Contents

Figure S1. A TEM image and histogram of the gold nanoparticles...2
Figure S2. A summary graph and spectra of the 280nm absorbance at varying levels of irradiation …………………......2
Figure S3. The individual measurements for each standard condition measured 2
Figure S4. STEM images of the nanoparticles at irradiation levels of 0 and 80 Gy 3
Figure S5. STEM images of the nanoparticles loaded with Irinotecan ………...3
Figure S6. Loading characteristics of the Zein and hybrid nanoparticles 4
Figure S7. Irinotecan fluorescent characteristics at different levels of irradiation 4
Figure S8. Standard characteristics of the hybrid particles at 20 Gys administered as a single dose or fractioned.........5
Figure S9. Standard characteristics of the Zein particles at 20 Gys administered as a single dose or fractioned............5

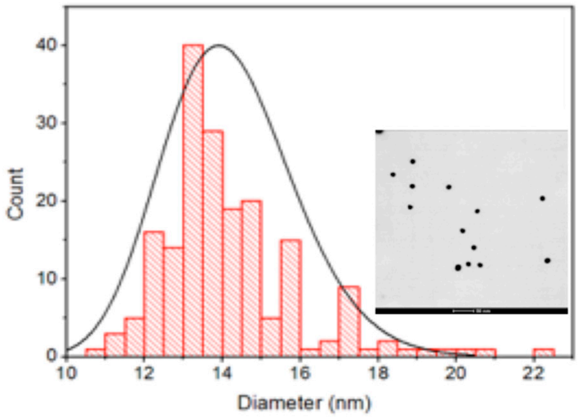

Figure S1. A histogram and TEM image of the 13nm gold nanoparticles used to make ZAuNPs.

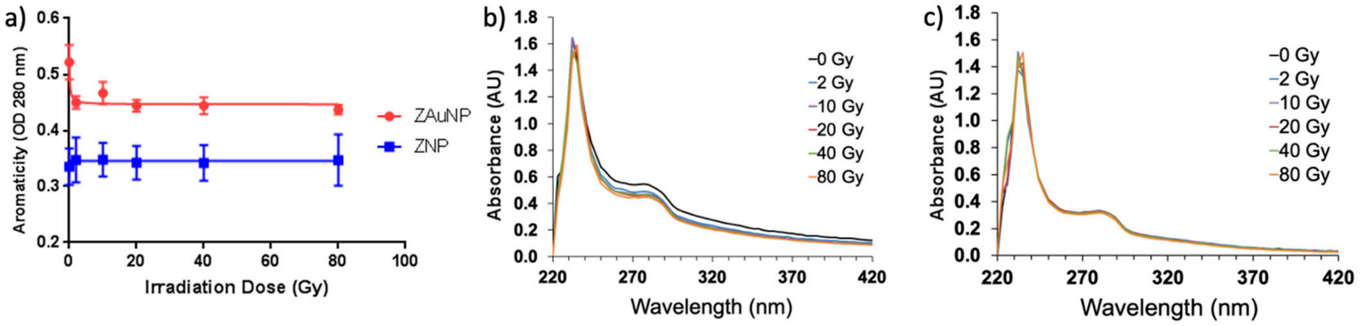

Figure S2. 280nm absorbance data of Zein nanoparticles (ZNPs) and Zein gold nanoparticle hybrids (ZAuNPs) at varying levels of irradiation. **(a)** A summary graph of the ZNPs and ZAuNPs at varying levels of irradiation. **(b)** UV-Spectra of the ZAuNPs at varying levels of irradiation. **(c)** UV-Spectra of the ZNPs at varying levels of irradiation.

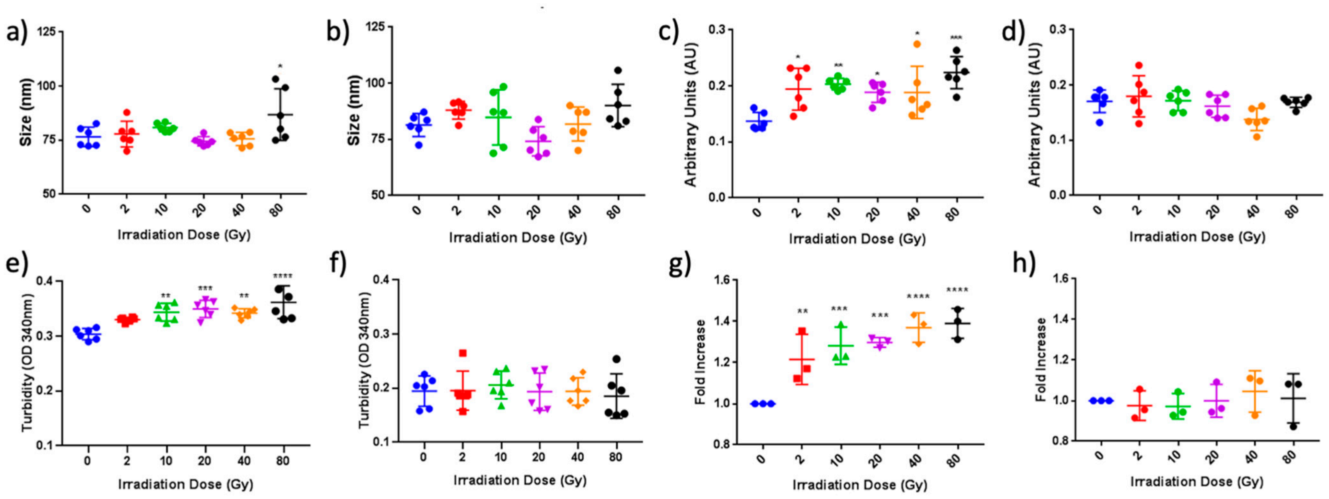

Figure S3. Individual measurements of Zein nanoparticles (ZNPs) and Zein gold nanoparticle hybrids (ZAuNPs) for size, polydispersity, turbidity and aggregation at varying levels of irradiation exposure. Each dose was indicated as statistically significant relative to the 0 Gy control when applicable. **(a)** ZAuNP size after varying levels of irradiation exposure **(b)** ZNP size after varying levels of irradiation exposure. **(c)** ZAuNP polydispersity after varying levels of irradiation exposure **(d)** ZNP polydispersity after varying levels of irradiation exposure. **(e)** ZAuNP turbidity after varying levels of irradiation exposure **(f)** ZNP turbidity after varying levels of irradiation exposure. **(g)** ZAuNP aggregation after varying levels of irradiation exposure **(h)** ZNP aggregation after varying levels of irradiation exposure.

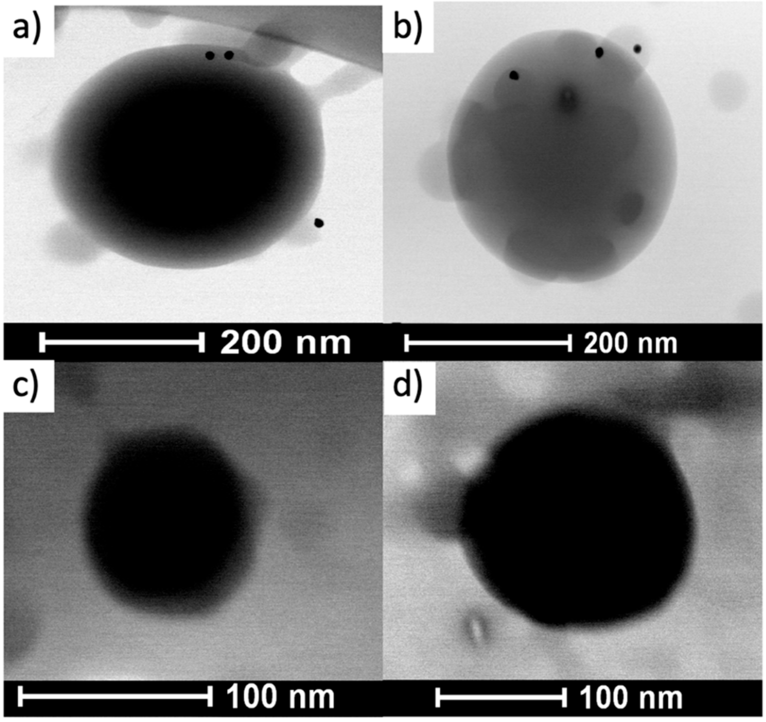

Figure S4. Depicts brightfield STEM images of Zein nanoparticles (ZNP) and Zein gold nanoparticle hybrids (ZAuNPs). (a) A brightfield image of a ZAuNP without irradiation exposure. (b) A brightfield image of a ZAuNP after 80 Gy irradiation. (c) A brightfield image of a ZNP without irradiation exposure. (d) A brightfield image of a ZNP after 80 Gy Irradiation.

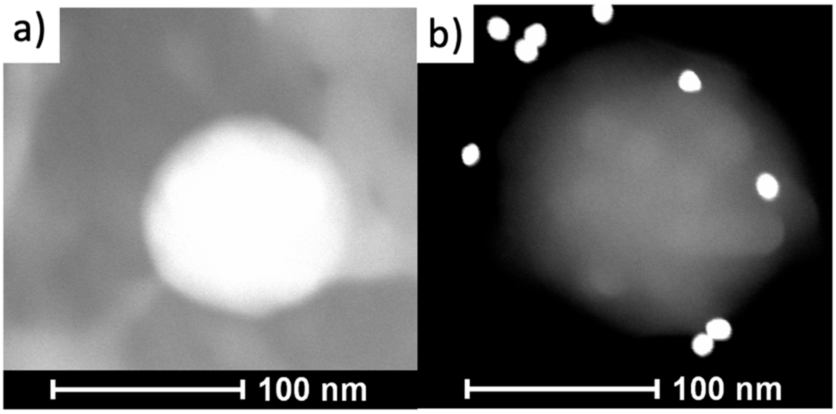

Figure S5. Darkfield STEM images of the Zein nanoparticles (ZNPs) and Zein gold hybrid particles (ZAuNPs) with Irinotecan **(a)** A darkfield image of a ZNP loaded with 50 μg/mL Irinotecan. **(b)** A darkfield image of a ZAuNP loaded with 50 μg/mL Irinotecan.

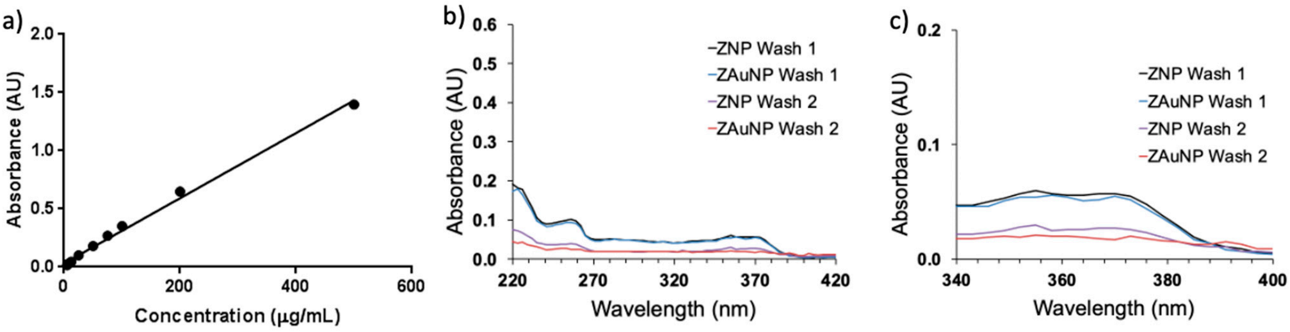

Figure S6. Loading characteristics of the Zein (ZNP) and Zein hybrid (ZAuNP) nanoparticles **(a)** Irinotecan absorbance calibration curve using Nanodrop-3500. **(b)** 220nm to 420nm UV-spectra of ZNP and ZAuNP 50 μg/mL formulation supernatants undergoing both washing steps. **(c)** 340nm to 400nm UV-spectra of ZNP and ZAuNP 50 μg/mL formulation supernatants undergoing both washing steps.

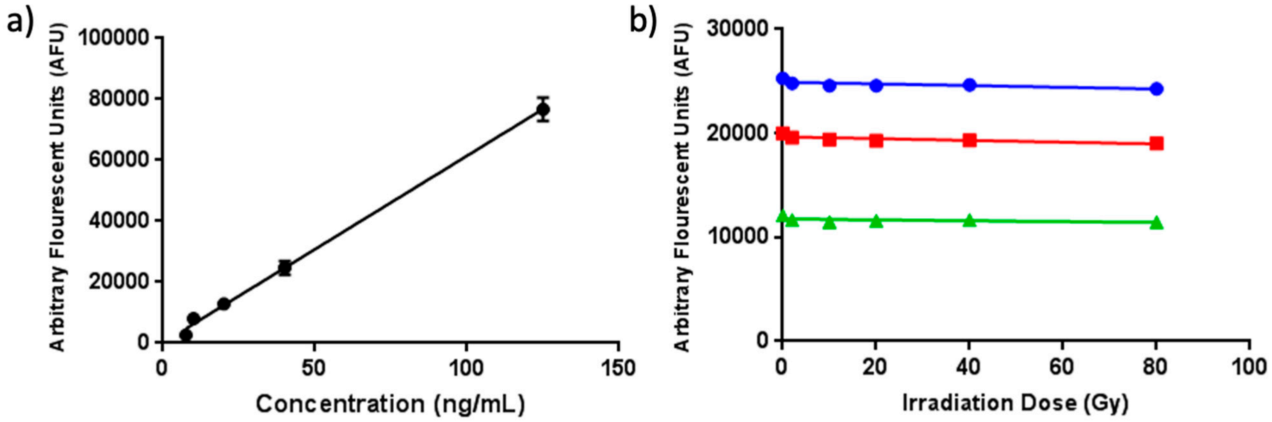

Figure S7. Irinotecan fluorescent characteristics at different levels of irradiation **(a)** Irinotecan fluorescent calibration curve using CLARIOStar plate reader at a gain of 2200. **(b)** Fluorescence of Irinotecan (gain 700) at varying concentrations (blue 37.5; red 25.0; green 12.5 μg/mL) pre- and post- irradiation exposure.

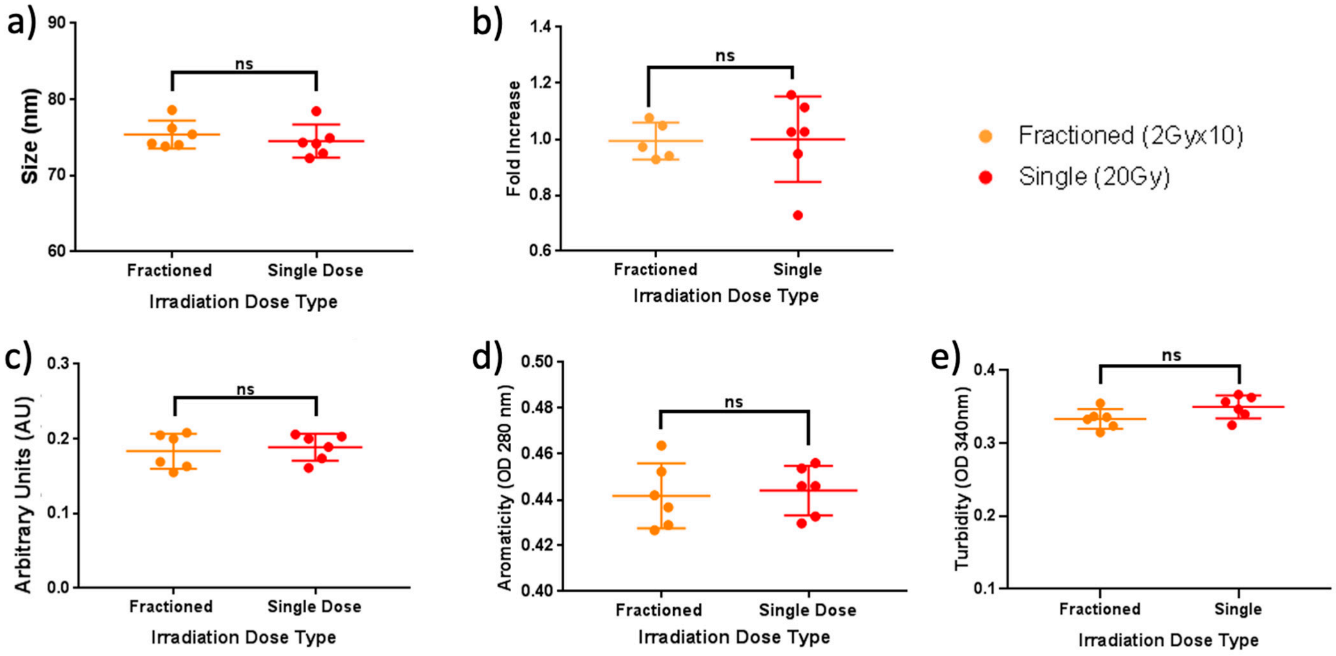

Figure S8. Standard characteristics of the Zein gold hybrid particles (ZAuNP) at 20 Gys administered as a single dose or fractioned **(a)** ZAuNP size post 20 Gy irradiation administered as a single or fractioned dosing scheme. **(b)** ZAuNP aggregation post 20 Gy irradiation administered as a single or fractioned dosing scheme. Normalized to single dosing scheme. **(c)** ZAuNP polydispersity post 20 Gy irradiation administered as a single or fractioned dosing scheme. **(d)** ZAuNP 280 nm absorbance post 20 Gy irradiation administered as a single or fractioned dosing scheme. **(e)** ZAuNP 340 nm absorbance post 20 Gy irradiation administered as a single or fractioned dosing scheme. ns, no significant difference.

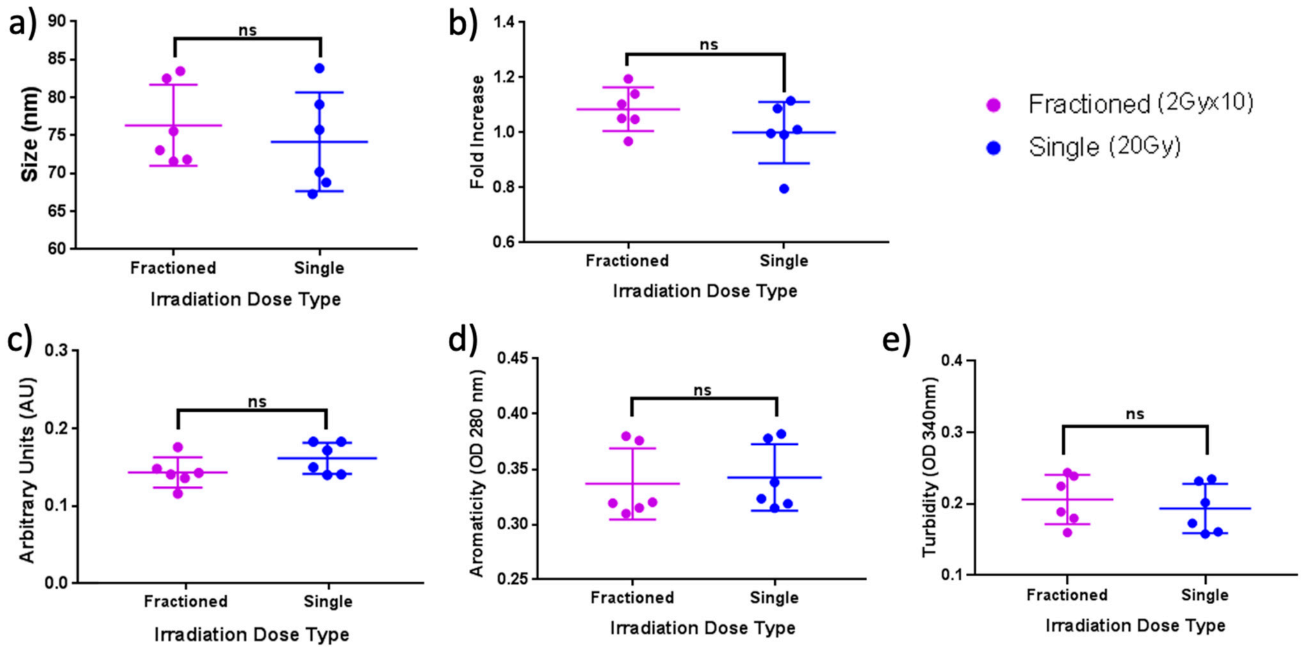

Figure S9. Standard characteristics of the Zein particles at 20 Gys administered as a single dose or fractioned **(a)** ZNP size post 20 Gy irradiation administered as a single or fractioned dosing scheme. **(b)** ZNP aggregation post 20 Gy irradiation administered as a single or fractioned dosing scheme. Normalized to single dosing scheme. **(c)** ZNP polydispersity post 20 Gy irradiation administered as a single or fractioned dosing scheme. **(d)** ZNP 280 nm absorbance post 20 Gy irradiation administered as a single or fractioned dosing scheme. **(e)** ZNP 340 nm absorbance post 20 Gy irradiation administered as a single or fractioned dosing scheme. ns, no significant difference.
